# Development and validation of a radiomics model based on T2WI images for preoperative prediction of microsatellite instability status in rectal cancer: Study Protocol Clinical Trial (SPIRIT Compliant)

**DOI:** 10.1097/MD.0000000000019428

**Published:** 2020-03-06

**Authors:** Zixing Huang, Wei Zhang, Du He, Xing Cui, Song Tian, Hongkun Yin, Bin Song

**Affiliations:** a Department of Radiology, West China Hospital, Sichuan University, Chengdu; b Department of Radiology, Sichuan Provincial Corps Hospital, Chinese People’ s Armed Police Forces, Leshan; c Department of Pathology, West China Hospital, Sichuan University, Chengdu; d Institute of Advanced Research, Infervision, Beijing, China.

**Keywords:** microsatellite instability, MRI, radiomics, rectal cancer, retrospective study

## Abstract

**Introduction:**

Globally, colorectal cancer (CRC) is the third most commonly diagnosed cancer in males and the second in females. Rectal cancer (RC) accounts for about 28% of all newly diagnosed CRC cases. The treatment of choice for locally advanced RC is a combination of surgical resection and chemotherapy and/or radiotherapy. These patients can potentially be cured, but the clinical outcome depends on the tumor biology. Microsatellite instability (MSI) is an important biomarker in CRC, with crucial diagnostic, prognostic, and predictive implications. It is important to develop a noninvasive, repeatable, and reproducible method to reflect the microsatellite status. Magnetic resonance imaging (MRI) has been recommended as the preferred imaging examination for RC in clinical practice by both the National Comprehensive Cancer Network and the European Society for Medical Oncology guidelines. T2WI is the core sequence of MRI scanning protocol for RC. Radiomics, the high-throughput mining of quantitative image features from standard-of-care medical imaging that enables data to be extracted and applied within clinical-decision support systems to improve diagnostic, prognostic, and predictive accuracy, is gaining importance in cancer research.

We proposed a hypothesis: A simple radiomics model based on only T2WI images can accurately evaluate the MSI status of RC preoperatively.

**Objective:**

To develop a radiomics model based on T2WI images for accurate preoperative diagnosis the MSI status of RC.

**Method:**

All patients with RC were retrospectively enrolled. The dataset was randomly split into training cohort (70% of all patients) and testing cohort (30% of all patients). The radiomics features will be extracted from T2WI–MR images of the entire primary tumor region. Least absolute shrinkage and selection operator was used to select the most predictive radiomics features. Logistic regression models were constructed in the training/validation cohort to discriminate the MSI status using clinical factors, radiomics features, or their integration. The diagnostic performance of these 3 models was evaluated in the testing cohort based on their area under the curve, sensitivity, specificity, and accuracy.

**Discussion:**

This study will help us know whether radiomics model based on T2WI images to preoperative identify MSI status of RC.

## 1 Introduction

Globally, colorectal cancer (CRC) is the third most commonly diagnosed cancer in males and the second in females, with 1.8 million new cases and almost 861,000 deaths in 2018.^[1]^ Rectal cancer (RC) accounts for about 28% of all newly diagnosed CRC cases.^[2]^ The treatment of choice for locally advanced RC is a combination of surgical resection and chemotherapy and/or radiotherapy. These patients can potentially be cured, but the clinical outcome depends on the tumor biology.^[3]^

Microsatellite instability (MSI) is an important biomarker in CRC, with crucial diagnostic, prognostic, and predictive implications. MSI-high (MSI-H) status is associated with a better prognosis in early-stage CRC and a lack of benefit from adjuvant treatment with 5-fluorouracil in stage II disease, and these patients have shown a significantly better prognosis than those characterized by microsatellite stability (MSS).^[4]^ More recently, MSI has emerged as a predictor of sensitivity to immunotherapy-based treatments.^[4,5]^ Therefore, MSI testing is recommended by the National Comprehensive Cancer Network (NCCN) guidelines for all patients with stage II RC,^[6]^ and the European Society for Medical Oncology (ESMO) also recommended MSI testing for cancer immunotherapy in 2019.^[7]^ Preoperative evaluation of MSI status can be performed only by biopsy. However, first, tumors have spatial and temporal heterogeneities,^[8]^ the result of MSI testing may vary depend on where and when the specimen are obtained. Second, The risks of invasive sampling and potential complications. Therefore, it is important to develop a noninvasive, repeatable, and reproducible method to reflect the microsatellite status.

The conversion of digital medical images into mineable high-dimensional data, a process that is known as radiomics, is motivated by the concept that biomedical images contain information that reflects underlying pathophysiology and that these relationships can be revealed via quantitative image analyses.^[9]^ Recent studies of RC have showed encouraging evidence that radiomics can be applied to predict characteristics including therapeutic responses,^[10,11]^ lymph node metastasis,^[12,13]^ and Kirsten sarcoma viral oncogene homologue mutations.^[14,15]^ And, the radiomics based on computed tomography images can predict the MSI status of CRC was reported in 2 recent studies.^[16,17]^ Magnetic resonance imaging (MRI) has been recommended as the preferred imaging examination for RC in clinical practice by both NCCN^[18]^ and ESMO^[19]^ guidelines. T2WI is the core sequence of MRI scanning protocol for RC.^[20]^

We proposed a hypothesis: a simple radiomics model based on only T2WI images can accurately evaluate the MSI status of RC preoperatively.

## 2 Participants and methods

### 2.1 Study aims

The aim of this study is to develop a radiomics model based on T2WI images for accurate preoperative diagnosis the MSI status of RC.

### 2.2 Study design/setting

This is a single-center, retrospective, cross-sectional clinical study. The T2WI data were anonymous.

### 2.3 Study registration

This clinical trial has been registered on the Chinese Clinical Trial Registry (www.chictr.org.cn), and the registration number is ChiCTR2000029362.

### 2.4 Eligibility criteria (Fig. 1)

The patients with RC underwent surgery at our hospital from January 2016 to May 2019 were retrospectively collected.

**Figure 1:**
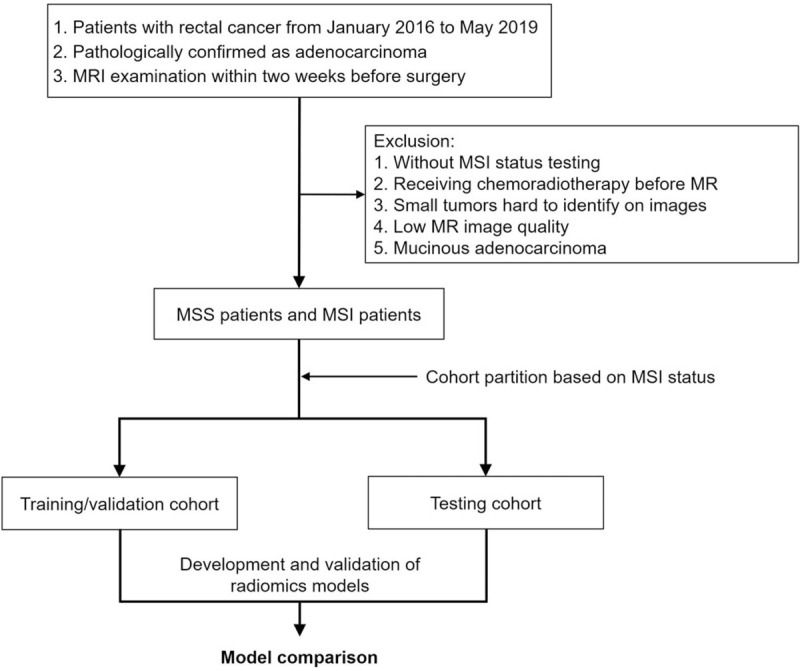
Flowchart of the patient selection and patient exclusion.

#### 2.4.1 Inclusion criteria

(1) Pathologically confirmed as adenocarcinoma.
(2) MRI within 2 weeks before surgery.

#### 2.4.2 Exclusion criteria

(1) Without MSI evaluation.
(2) Receiving chemoradiotherapy before MRI.
(3) Small tumors (<5 mm) difficult to identify on images.
(4) Insufficient T2WI quality to draw the region of interest (ROI) such as obvious motion artifact caused by respiration or intestinal peristalsis.
(5) Mucinous adenocarcinoma.

### 2.5 Intervention

This study is a retrospective analysis of MRI T2WI images, with no intervention measures for patients.

### 2.6 Radiomics analysis (Fig. 2)

#### 2.6.1 Clinical and pathological data

Clinical data include: age, sex, carcinoembryonic antigen, and carbohydrate antigen 19-9.

**Figure 2:**
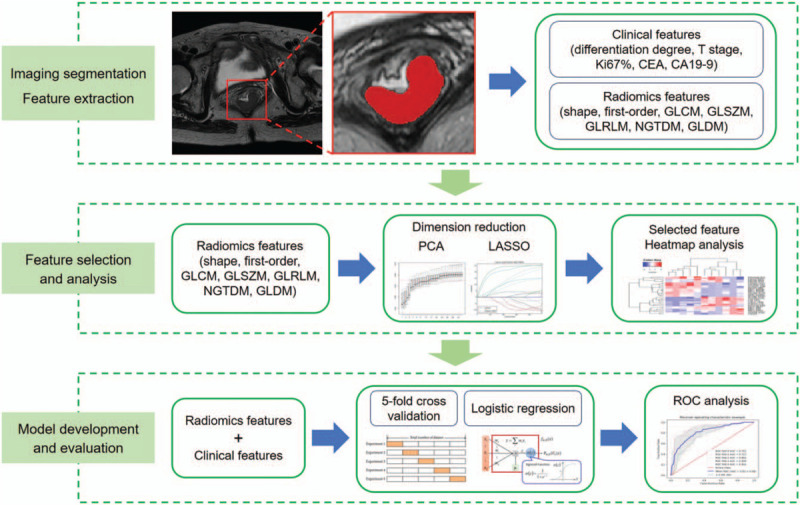
The radiomics analysis framework in this study.

Pathological data include: tumor differentiation degree, T stage, Ki-67 expression, and MSI status. Considering the Bethesda guidelines for CRCs suggesting that the terms MSI-H or MSI-low (MSI-L) be discontinued and MSI-L tumors be included with MSS tumors, the MSI-H was regarded as MSI and MSI-L was regarded as MSS in this study.

#### 2.6.2 MRI image acquisition and segmentation

All patients underwent MRI scan following protocol for RC within 2 weeks before surgery or biopsy. By using the ITK-SNAP software (v3.6.0, http://www.itksnap.org), the tumor region was manually segmented volumetrically by a radiologist with more than 10 years of experience in abdominal imaging on all oblique axial (angulated perpendicular to the long axis of the rectal tumor) of T2WI slices. The necrosis area, intraluminal air regions, and uninvaded rectal wall were excluded from the ROIs with caution. In case that the boundary was uncertain, a final decision was made by another radiologist with 20 years of experience in abdominal diagnosis.

#### 2.6.3 Feature extraction

To achieve a standard normal distribution of image intensities, MRI scans for each patient were normalized with *z*-scores. Feature extraction was performed using an open-source Python package (PyRadiomics version 2.1.2, https://github.com/Radiomics/pyradiomics), radiomics features including shape, first-order intensity statistics, and texture features were automatically extracted from the manually segmented tumor regions.

#### 2.6.4 Inter- and intraobserver reproducibility evaluation

To evaluate the reproducibility and accuracy of image segmentation and feature extraction, we randomly selected 50 MR scans and performed double-blind manual segmentation by 2 radiologists repeatly. The radiomics features from the 50 paired segmentation results were automatically extracted, and intraclass correlation coefficients (ICC) was used to access the inter- and intraobserver agreement.

#### 2.6.5 Feature selection

The feature extraction and selection process were performed using Python 3.6.0 (www.python.org). Only the extracted radiomics features with good reproducibility (ICC >0.75) were selected for further analysis. To prevent overfitting of the model, the variance threshold approach (scikit-learn 0.22.1, sklearn. feature_selection module) was used to remove all features whose variance did not meet a certain threshold based on the training queue. The least absolute shrinkage and selection operator algorithm was used to select the key radiomics features that were the most closely associated with determination of MSI status in RC.

#### 2.6.6 Development of the radiomics models

We used multivariable logistic regression analysis to develop 3 models: radiomics clinicopathological model, radiomics imaging model, and combined model integrating radiomics imaging and clinical information. Selected radiomics feature values were standardized to the (0, 1) interval to avoid redundancies and inconsistencies. The radiomics clinicopathological model was developed with clinical parameters. The radiomics model was constructed with selected features. The combined model was built with both selected radiomics features and clinical parameters. The 5-fold cross validation approach was used in the training/validation cohort to better train our model and build it more robustly. The development and validation of the radiomics models were performed with the InferScholar platform version 3.1 (InferVision, China).

### 2.7 Outcome measure

The radiomics model which identify potential differences between RC without MSI-H group and RC with MSI-H group will be the outcome measure.

### 2.8 Data collection and management

Data will be processed anonymously, omitting the information that can identify the participant's individual identity. Strict safety and confidentiality measures will be established in the archives of clinical trial institutions.

### 2.9 Sample size calculation

We calculated the number of patients needed considering the epidemiology of MSI in RC and previous report by Wu et al.^[21]^ Test for 1 receiver operating characteristic (ROC) curve analysis showed that a sample of 33 from the MSI group and 297 from the MSS group could achieve 95% power to detect a difference of the null hypothesis (area under the curve [AUC] = 0.70) and the alternative hypothesis (AUC = 0.85), which was calculated by PASS 2019 (NCSS Statistical Software).

### 2.10 Statistical analysis

The ROC curve analysis was conducted, and AUC, accuracy, sensitivity, and specificity were calculated to evaluate the diagnostic performance of each model. Mann–Whitney *U* test and Chi-squared test or Fisher exact test were performed to evaluate the differences in variables with a continuous distribution across categories and the association among categorical variables, respectively. A 2-sided *P*-value of <.05 was considered as statistically significant. Prism 5 for Windows (Version 5.01) and MedCalc (Version 18.11.3) were used for statistical analysis.

### 2.11 Ethics and dissemination

This retrospective study was approved by the Biomedical Research Ethics Committee of West China Hospital of Sichuan University (No. 2019-1159), and the requirement for informed consent was waived. To protect privacy of participants, all of private information were anonymous. The results will be published in a peer-reviewed journal and will be disseminated electronically and in print regardless of results. The authors report no conflicts of interest.

## 3 Discussion

Radiomics, the high-throughput mining of quantitative image features from standard-of-care medical imaging that enables data to be extracted and applied within clinical-decision support systems to improve diagnostic, prognostic, and predictive accuracy, is gaining importance in cancer research. Radiomic analysis exploits sophisticated image analysis tools and the rapid development and validation of medical imaging data that uses image-based signatures for precision diagnosis and treatment, providing a powerful tool in modern medicine.^[22]^ In this study, we will develop a radiomics model based on T2WI images to preoperative identify MSI status of RC. The model may provide a preoperative, and easy-to-use tool for MSI status diagnosis, which can help oncologists make better treatment choices for RC patients.

Our study has several limitations. We used the retrospective datasets to develop radiomics model, of which some clinical factors were not initially available on account of incomplete data. The ROIs were delineated in 1 single slice (2D), which might not be representative of the entire tumor. As a single-center retrospective study, the model needs to be validated by future multicenter studies to evaluate or improve the performance.

We hope that the radiomics model can be an excellent prediction performance on MSI status of RC and may have significant clinical implications on noninvasive assessment of MSI status for RC.

## Author contributions

**Conceptualization:** Zixing Huang, Wei Zhang, Bin Song.

**Data curation:** Wei Zhang, Du He.

**Methodology:** Zixing Huang, Hongkun Yin.

**Resources:** Bin Song, Du He.

**Radiomics analysis:** Hongkun Yin, Song Tian, Xing Cui.

**Supervision:** Bin Song.

**Writing – original draft:** Zixing Huang, Wei Zhang, Hongkun Yin.

**Writing – review and editing:** Zixing Huang, Bin Song.
